# Nutritional considerations during prolonged exposure to a confined, hyperbaric, hyperoxic environment: recommendations for saturation divers

**DOI:** 10.1186/s13728-015-0042-9

**Published:** 2016-01-07

**Authors:** S. K. Deb, P. A. Swinton, E. Dolan

**Affiliations:** School of Health Sciences, Robert Gordon University, Aberdeen, AB10 7QG UK; Department of Sport and Physical Activity, Edgehill University, Ormskirk, Lancashire UK; Laboratory of Applied Nutrition and Metabolism, School of Physical Education and Sport, University of Sao Paulo, São Paulo, Brazil

**Keywords:** Saturation diving, Hyperbaria, Hyperoxia, Confinement, Nutrition

## Abstract

Saturation diving is an occupation that involves prolonged exposure to a confined, hyperoxic, hyperbaric environment. The unique and extreme environment is thought to result in disruption to physiological and metabolic homeostasis, which may impact human health and performance. Appropriate nutritional intake has the potential to alleviate and/or support many of these physiological and metabolic concerns, whilst enhancing health and performance in saturation divers. Therefore, the purpose of this review is to identify the physiological and practical challenges of saturation diving and consequently provide evidence-based nutritional recommendations for saturation divers to promote health and performance within this challenging environment. Saturation diving has a high-energy demand, with an energy intake of between 44 and 52 kcal/kg body mass per day recommended, dependent on intensity and duration of underwater activity. The macronutrient composition of dietary intake is in accordance with the current Institute of Medicine guidelines at 45–65 % and 20–35 % of total energy intake for carbohydrate and fat intake, respectively. A minimum daily protein intake of 1.3 g/kg body mass is recommended to facilitate body composition maintenance. Macronutrient intake between individuals should, however, be dictated by personal preference to support the attainment of an energy balance. A varied diet high in fruit and vegetables is highly recommended for the provision of sufficient micronutrients to support physiological processes, such as vitamin B12 and folate intake to facilitate red blood cell production. Antioxidants, such as vitamin C and E, are also recommended to reduce oxidised molecules, e.g. free radicals, whilst selenium and zinc intake may be beneficial to reinforce endogenous antioxidant reserves. In addition, tailored hydration and carbohydrate fueling strategies for underwater work are also advised.

## Background

**Fig. 1:**
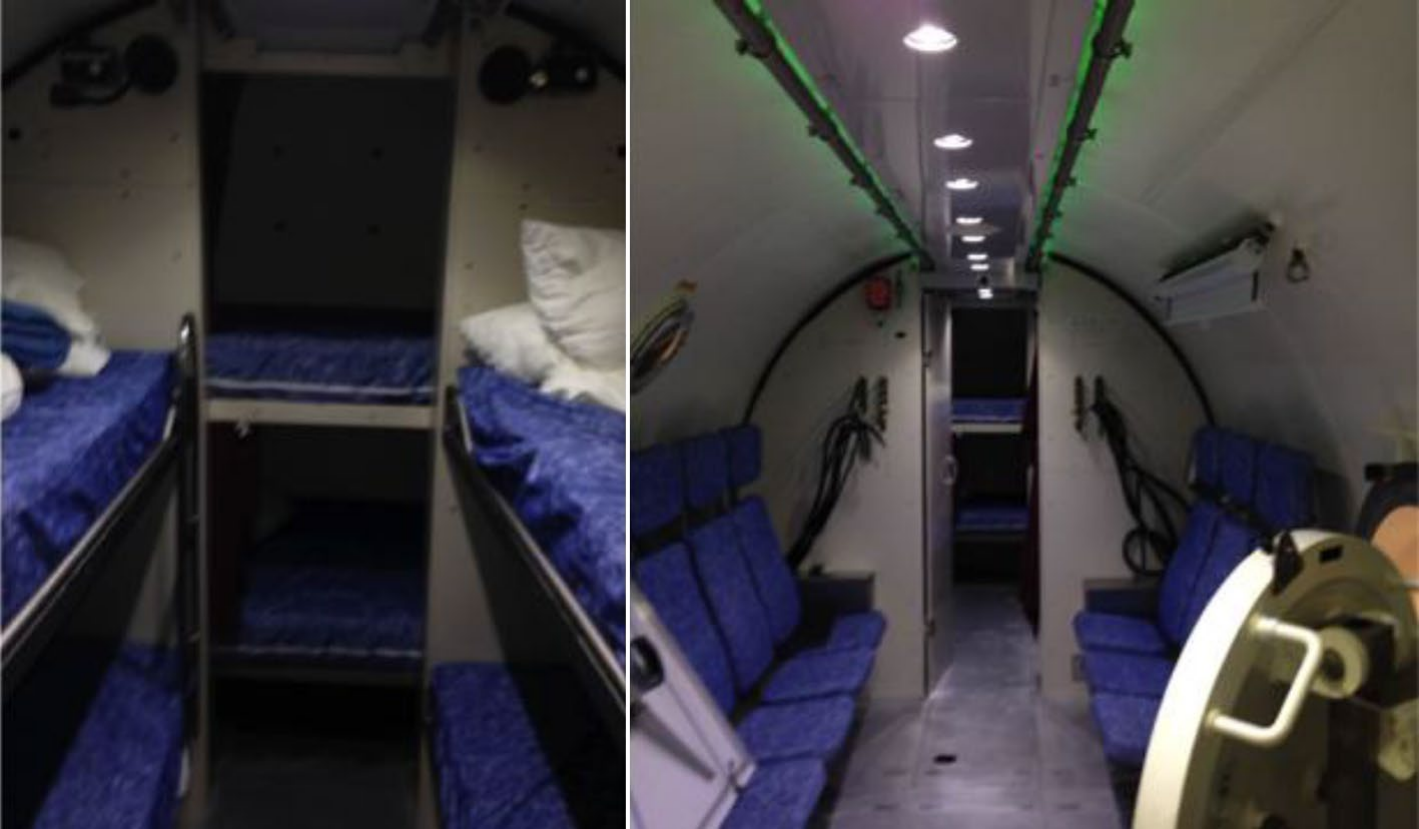
Image on the *left* shows the sleeping quarters, shared between 6 divers. The image on the *right* shows the full length of the living quarters shared by saturation divers for up to 28 consecutive days

Saturation diving is a unique and challenging occupation, which exposes the body to a range of extreme environmental and physiological stressors [1–3]. This form of diving allows greater depths to be attained and for longer periods than can be achieved with other forms of diving (e.g. breath hold or bounce diving). Accordingly, saturation diving involves habitation within a dry hyperbaric chamber located on a support vessel (see Fig. 1), which is pressurised to an ambient pressure equivalent to the depth of the underwater immersion. Divers remain within this pressurised chamber for extended periods (e.g. up to twenty-eight days in the North Sea Energy Sector), thereby allowing numerous underwater excursions without the requirement of a prolonged decompression period following every immersion [1, 2].

The hyperbaric conditions associated with saturation diving represents the linear relationship between increasing atmospheric pressure and sea depth [1]. Chronic exposure to this unique environment appears to disturb physiological and metabolic homeostasis and has been associated with a range of adverse health and performance-related outcomes [2, 3]. In particular, the environmental conditions of saturation diving have been reported to perturb fluid balance [4–6], redox homeostasis [7–9], immunological function [10] and haematological variables [11]. Furthermore, significant reductions in body mass are commonly reported, specifically muscle mass [12, 13], which may be associated with the attenuation of whole body protein synthesis [14], increased basal metabolism [15] and altered metabolic fuel utilisation [16, 17]. In addition, saturation diving involves confinement within a relatively small space for extended periods, rendering maintenance of usual physical activity habits difficult [18].

Appropriate nutritional strategies have the potential to alleviate many of these physiological concerns; however, limited guidance related to nutritional recommendations for this population is currently available. The purpose of this review, therefore, is to explore the physiological and practical challenges associated with this occupation. These challenges will then be considered within the context of current nutrition guidelines for general and athletic populations, in order to establish specific nutritional recommendations to optimise health and performance of individuals in this unique and challenging occupation. It is beyond the scope of this review to provide an in-depth review of the physical characteristics or physiological and pathophysiological concerns of saturation diving. Readers are directed to alternative reviews for such information [1–3].

## Saturation diving

Saturation diving practices differ based on location and sector but can typically extend for periods of up to twenty-eight days within a hyperbaric, mixed gas atmosphere. The compressive forces on the gaseous molecules from hyperbaria result in increased density of ambient air and increased partial pressure of oxygen (PPO_2_). Typically, the gas mixtures used during a saturation dive comprise a combination of oxygen and helium and/or nitrogen. These latter two gases are known as inert gases, which are deemed to metabolically inactive and thereby purported to have a minimal effect on physiological systems [1–3]. Due to the distinct atmospheric composition in the hyperbaric chamber, saturation divers are exposed to three discrete sequential phases, which are commonly referred to as, ‘compression’, ‘bottom time’ and ‘decompression’. The purpose of ‘compression’ is to attain a dynamic equilibrium between the body and the prescribed ambient atmospheric characteristics, which is a gradual process that takes a number of hours. Saturation divers remain in this equilibrated state during ‘bottom time’, at which, assuming there is no additional change in depth, divers can live freely within the chamber and participate in underwater excursions up to and including the depths that correspond to the chamber pressure. During underwater excursions, which are often termed as ‘lockout’, saturation divers are immersed underwater in order to conduct a range of manual tasks, such as welding. This period can usually last up to 8 h, six of which are typically spent within the water and the other two being transferred to and from the accommodation area.

‘Decompression’ is the final phase, during which saturation divers are returned to the terrestrial atmospheric pressures through the gradual reduction of ambient pressure. As with compression, the body equilibrates with the altered ambient atmosphere, which occurs through exhalation of compressed gases from body tissues through cardiorespiratory circulation. This process is associated with potentially adverse consequences, and an increased risk of developing decompression sickness (DCS), which can cause muscle and joint pain, nausea and paralysis. Manifestations of DCS occur via the formation of microbubbles along with associated inflammatory and immunological challenges [19]. In order to reduce the risk for development of DCS, decompression is a slow and tightly controlled process, with the duration’s dependent upon the depth descended and decompression rate utilised. Decompression times of 5–6 days are not uncommon. Throughout the ‘decompression’, saturation divers do not participate in any subsea activities and are limited to the confines of the saturation chamber.

## Nutritional recommendations

The effect of dietary intake on metabolic and physiological processes is vast, with the application of personalised nutritional strategies deemed to be most effective in promoting performance and health. Accordingly, the current review aims to synthesise the available literature related to the physiological responses to saturation diving, and to consider these in relation to commonly accepted nutritional principles [20–22]. Recommendations have been made within 4 key categories including: (1) maintenance of energy balance; (2) macronutrient composition of dietary intake; (3) micronutrient requirements and (4) hydration. These recommendations are made based on the available evidence, but remain cognisant of the practical challenges associated with achieving appropriate nutritional intake in this unique environment.

### Energy balance

Consistent reports of body mass loss while in saturation [12, 13, 23] suggests that divers may be in an energy-deficient state, and this loss includes muscle mass [13]. Chronic energy deficit while in saturation may contribute to a number of adverse health outcomes, including immunosuppression and compromised bone health (10, 2). It has been suggested that energy deficiency during saturation may, at least in part, be due to an increased energy cost of activities performed under saturation, relative to the energy cost of those same activities under usual environmental conditions [15]. For example, helium (which is often used as the inert gas required to create the hyperbaric environment) possesses a conductivity six times greater than oxygen [3], which increases heat exchange from the body, therefore increasing the energy cost of thermoregulation. In addition, hyperbaria results in a greater gas density thereby increasing airway resistance and subsequently the energy cost of respiration [24]. Using doubly labelled water, a significant increase from 3105 ± 95 to 3534 ± 119 kcal was reported when comparing energy expenditure at the surface and 317 m depth, respectively, in US navy saturation divers [15]. A comparison between 50 and 317 m revealed no significant difference in energy expenditure, indicating the increase in energy expenditure may be due to the composition of the gaseous mix as opposed to pressure differences associated with depth. Further research by Busch-Stockfisch and Bohlen [13] indirectly supported this notion of increased energy expenditure though comparison of nutritional intake and changes in body composition in operational saturation divers. This study reported a mean 1.98 kg loss in muscle mass and 2.65 kg loss in total body mass over the course of an extended operational saturation dive between 30 and 44 days, despite dietary intakes remaining comparable with onshore intakes.

#### Energy intake recommendations

Paciorek [25] calculated that saturation divers required approximately 53 kcal/kg body mass (BM) a day. This calculation was based on maintenance of body mass of six divers during a 17 day working dive at 350 m which involved 6 days of 8 h working shifts, a working pattern which would not be uncommon with current operational practices. Using Paciorek's [25] proposed figure, an 80 kg diver would require 4249 kcal daily, a figure that would seem appropriate considering the work of Seale et al. [15] who reported expenditure of 3534 ± 119 kcal in 79.5 ± 2.5 kg navy divers at 317 m during a dry simulated dive (i.e. no underwater work was performed). Using the available evidence, the proposed lower limit of required energy intake is 44 kcal/kg BM a day, which is based on a 79.5 kg diver requiring 3534 kcal on average as reported by Seal et al. [15]. Therefore, saturation divers should aim to consume between 44 and 53 kcal/kg BM a day dependent on the duration and intensity of work performed on a given day. As energy requirements will vary daily amongst individuals, divers should monitor their daily energy intake against subjective feelings of fatigue, energy and hunger to assist in achieving an energy balance.

### Macronutrient composition

#### Carbohydrates and dietary fats

Research regarding the utilisation of metabolic substrates at rest and during physical activity during hyperbaria or hyperoxia is sparse [17, 26, 27], although the available evidence supports the notion of increased fat utilisation. It was deemed important to address the issue of substrate utilisation due to the potential influence on macronutrient recommendations during work and rest periods. For example, research conducted under hypoxia has demonstrated altered substrate utilisation [28], leading to tailored nutritional recommendations for increased carbohydrate ingestion. It is documented that exercise under acute hyperoxic conditions lowers the respiratory exchange ratio (RER) of exercise compared to normoxia [17, 26, 27] indicating increased fat utilisation. An earlier study by Dressendorfer et al. [16] conducted under hyperbaric conditions assessed maximal oxygen uptake in three different conditions, including an atmosphere equivalent to the ambient surface air, a hyperoxic heliox atmosphere and a normoxic heliox atmosphere during a seventeen-day simulated saturation dive. Although the main purpose of the study does not provide relevant data for the purposes of this section, further analysis of the presented raw data of the four subjects does. The Vo_2_ and VCo_2_ data provided during submaximal exercise allowed calculation of fat and carbohydrate oxidation using stoichiometric equations [29]. The analysis revealed that energy expenditure was greater in both heliox atmospheres, as supported by subsequent research [15]. In addition, carbohydrate oxidation within both heliox conditions was over twofold smaller (0.83 vs. 1.70 g/min) and fat oxidation was four-fold greater (0.50 vs. 0.14 g/min) in hyperoxia compared with normoxia. These results suggest that chronic exposure to hyperoxia, irrespective of hyperbaria, results in increased fat oxidation. However, these data were taken from 5 min of submaximal exercise using the Douglas bag technique, which suffers from increased error when conducted in hyperoxia [30]. Therefore, these data should be used only as an indication of substrate use during saturation, while further research is required to better understand the metabolic implications of prolonged hyperoxic exposure.

#### Carbohydrate and dietary fat recommendations

The information presented on fuel utilisation under hyperoxia demonstrates a trend towards increased fat oxidation, but no study has directly assessed fuel use during an extended saturation dive either in the chamber or during an underwater excursion. Therefore, the current research may not be considered strong enough to provide recommendations that greatly deviate from general guidelines proposed by the Institute of Medicine (IOM) [20]. Current guidelines for dietary fat propose total intake should comprise 20–35 % of total calorie intake [20]. Due to the potential shift in fuel utilisation under hypoxia, which results in increased fat oxidation, we suggest saturation divers should consume dietary fats at the upper end of the range set out by the IOM. Furthermore, fat is more energy dense than carbohydrate (~9 vs. 4 kcal/g, respectively), therefore consuming a higher proportion of dietary fat may also facilitate saturation divers to meet the increased energy demand required under the heliox atmosphere. Catering provision on diving support vessels should endeavour to provide good dietary fat options on the menu so divers can consume higher intakes without compromising health. Table 1 provides examples of recommended dietary fat sources.

**Table 1:** Recommended sources of dietary fat which should be made available to saturation divers to attain the nutritional recommendations outlined for fat and total energy intake

Avocado	Flax seeds
Dairy (e.g. milk and yogurts)	Chia seeds
Nut butter (e.g. peanut/almond)	Coconut oil
Olives	Pumpkin seeds
Oily fish (e.g. mackerel and salmon)	Almonds
Hummus	Walnuts

Current carbohydrate recommendations for the general population are between 45 and 65 % of total calorie intake [20]. To allow sufficient intake of all three macronutrients and to allow for fat intake towards the top end of the recommended range, divers should be encouraged to target the low to mid carbohydrate intake of the current IOM guidelines. It should be made clear that we are not recommending a low-carbohydrate diet for saturation divers, but rather an appropriate balance of all three macronutrients to support metabolic function. Despite these suggestions, we believe that achieving energy balance is the most essential component of nutritional intake in this group in order to support physiological functions and maintain energy availability during lockout. Along with the absence of strong evidence on fuel use, it is suggested the appropriate balance of these two macronutrients should primarily be determined by the individual’s preferences, so to enable divers to achieve energy balance.

#### Protein metabolism

Knowledge of protein metabolism is important to provide guidelines on appropriate protein intakes, due to the implications on health and body composition [31, 32]. A series of simulated dives at various depths (200–600 m) revealed divers experienced reductions in lean body mass of between 1 and 5 kg over dives lasting 26–44 days [13]. This suggests divers may find themselves in a catabolic state, in which case, a higher protein intake is required to reduce skeletal muscle loss [33]. To the authors’ knowledge, only a single study has investigated whole body protein synthesis (WBPS) in this environment [14]. A significant twofold reduction in protein synthesis was reported between the surface and a simulated 45 m dive. Declines of this magnitude are typically associated with disease states, inadequate protein intakes, or negative energy balance [34]. However, in the study conducted by Conway et al. [14], subjects were healthy US navy divers who were in a positive nitrogen balance, consuming a protein rich diet equivalent to 1.5 g/kg BM a day throughout the trial. In addition, data from two adjunctive studies [15, 23] using the same sample revealed that the subjects also attained a balance between energy expenditure and intake. The results from these studies demonstrate that diminished WBPS may be attributed to dive conditions.

#### Protein recommendations

It appears appropriate to propose that under the unique environmental and physiological challenges, protein intake for saturation divers should be above the recommended daily allowance for the general population, which is currently 0.8 g/kg BM a day. An appropriate recommendation for this population would be a minimum of 1.3 g/kg BM a day, aligning with current recommendations to maximise muscle protein synthesis [35]. It may be conceivable that higher protein intakes are required to maximise WBPS under saturation based on the data reported by Conway et al. [14] but further research is required to define optimal protein requirements from a molecular perspective. Research in athletic populations suggests protein intake of between 1.8 and 2 g/kg BM a day would be appropriate to prevent muscle loss while in an energy-deficient state [35]. However, a main consideration when setting this protein intake was the contribution to achieving total energy requirements. Protein has high-thermogenic properties and promotes feeling of satiety, which will reduce appetite and subsequent energy intake [36] thus increasing the difficulty in achieving energy balance. Therefore, protein intake should be kept at a moderately increased level (1.3 g/kg) to help divers achieve a daily energy balance.

### Micronutrient requirements

Micronutrients are involved in various physiological functions in the body and are essential to the overall health of an individual. It is likely that if the higher energy intake described previously is achieved through a balanced and varied diet then the current IOM guidelines for micronutrient intakes will be met [20]. For saturation divers, the role of certain micronutrients may become even more relevant, particularly for issues related to bone health, oxidative stress and haematological processes, and these will be discussed throughout the forthcoming section.

#### Vitamin D

Brubakk et al. [2] identified bone health as one of the few documented long-term disease states linked with saturation diving. Bone is a nutritionally modulated tissue meaning dietary intake can have a significant impact on overall bone strength and structure [37]. Vitamin D has a pivotal role in regulating calcium homeostasis and therefore overall bone health. Vitamin D is also proposed to have implications on other physiological aspects such as immune function [38], insulin resistance [39] and physical performance [40]. We have focused on the role of vitamin D in these guidelines due to the probability that insufficient or deficient levels of serum 25[OH]D, a biomarker of vitamin D, may be prevalent amongst saturation divers due to lack of sunlight exposure within the saturation chamber.

Vitamin D is unique compared to other essential vitamins, predominantly as the main source is through ultraviolet B (UVB) radiation from sunlight as opposed to dietary intake [41]. According to some researchers, [42] in the absence of adequate sunlight exposure, it is unlikely that individuals can obtain sufficient vitamin D through diet alone. In saturation divers, this has obvious concerns as they are deprived of sunlight exposure for up to 28 days at a time. It appears reasonable to postulate that divers may have inadequate or deficient levels of serum 25[OH]D. Only one study has measured serum 25[OH]D concentrations, before and after a saturation dive lasting 14 days [7], during which serum 25[OH]D fell by 11 nmol L^−1^ by day 12 (from 92 ± 23 to 81 ± 18 nmol L^−1^) in a sample of six divers. If this trends were to continue over a full 28 day dive, concentrations could drop by more than 20 nmol L^−1^. Smith et al. [7] conducted the study in America, which may explain the high levels of baseline serum 25[OH]D.

#### Vitamin D recommendations

Due to the difficulty of obtaining sufficient vitamin D through dietary intake alone [42], along with the absence of UBV radiation, supplementation is deemed an appropriate strategy while in saturation. The aim for vitamin D recommendations should to be to maintain serum 25[OH]D > 50 nmol L^−1^, the current IOM threshold for vitamin D adequacy [43]. We recommend that saturation divers should receive regular blood analysis of vitamin D status so appropriate supplement strategies can be determined. Vitamin D is correlated with seasonal variations [43], so we suggest testing in the autumn and winter months when levels are likely to be at their lowest. Regardless of testing it would be prudent for divers to supplement whilst in the chamber. It has been proposed that supplementation with 2000 IU daily is sufficient to maintain serum 25[OH]D levels [44] whilst 4000 IU is defined as the upper tolerable limit by the IOM [45]. Therefore, we recommend that saturation divers can supplement between 2000 and 4000 IU safely when in saturation to maintain 25[OH]D concentrations. Supplementation can continue at this level onshore without any concern but may not be necessary, depending on the climate in the location divers reside, time of year, outdoor sunlight exposure and serum 25[OH]D test results.

#### Antioxidants

The term oxidative stress refers to a state caused by the generation of oxidised molecules such as reactive oxygen species (ROS) and free radicals that are greater than the ability of the body’s antioxidant system to reduce them, and may result in damage to cellular proteins, lipids and DNA. Research has implicated this imbalance between ROS and antioxidant reserves with the pathogenesis of various disorders, including cardiovascular and neurodegenerative diseases, diabetes and several types of cancer [46–48]. While intermittent exposure to oxidised particles may elicit a net positive adaptive response, chronic exposure to hyperoxia within the saturation chamber has been reported to elicit a greater magnitude of oxidative stress compared to the ambient surface atmosphere, resulting in increased lipid peroxidation and DNA damage [7, 8]. In addition to the reported oxidative damage, endogenous antioxidant enzyme activity has also been reported to be attenuated, specifically related to superoxide dismutase (SOD) activity [7–9]. Despite this, the full effect of saturation diving on redox physiology is yet to be ascertained, in particular other factors such as the chronic high pressure exposure and the effect of inert gas mixtures, which may contribute to oxidative stress [2]. It is however, unknown if the associated long-term health implications of chronic oxidative stress are prevalent in this population, as no epidemiological data exist [2]. Consumption of exogenous antioxidants can attenuate the magnitude of oxidative stress experienced under hyperoxic conditions [49]. Results from this study showed that supplementation of vitamin C (600 mg) and E (150 mg) diminished oxidative damage in the liver; however, to the authors’ knowledge, this has been the only study to investigate antioxidant supplementation in this environment [49].

#### Antioxidant recommendations

Exogenous antioxidants obtained through a healthy diet are associated with low levels of oxidative stress. A number of randomised controlled trials have shown that fruit and vegetable intake, which are antioxidant rich, can reduce markers of oxidative stress in healthy individuals [50–52]. Therefore, a varied diet high in fruit and vegetables is recommended during a dive. It is not known, however, if a varied dietary intake alone will be sufficient to attenuate oxidative damage during a saturation dive. Additional supplementation is not likely to cause any harm to individuals; therefore, supplementation of antioxidants at the same dosage used by Ikeda et al. [49] is deemed a safe and appropriate recommendation for saturation divers. Another consideration of redox homeostasis is the reduced endogenous antioxidant enzyme activity [7–9]; therefore, dietary intake to elevate both SOD and glutathione peroxidase may be prudent. Dietary intake can support these enzymes such as selenium intake to increase GPX activity [53] and zinc intake, which may elevate SOD [54]. It is prudent for saturation divers to meet the IOM guidelines on and offshore for selenium and zinc, which are 55 and 11 mg/day, respectively.

#### Vitamin B12, folate and iron

Haemoglobin concentrations throughout the course of a saturation dive have consistently been shown to decline [7, 9, 55, 56]. Nakabasy et al. [55] suggested the primary cause of reduced haemoglobin concentration is the attenuation of the rate of red blood cell production, which is also supported by the finding of reduced erythropoietin (EPO) activity during a dive [9]. In these guidelines, we focus on vitamin B12, folate and iron due to their involvement in haematological processes.

#### Vitamin B12 and folate recommendations

Vitamin B12 and folate are involved in the production red blood cells [57] and are linked to EPO activity [58, 59]. It is therefore deemed appropriate that saturation divers should consume sufficient levels of both nutrients to maintain normal circulating levels. Recommendations for vitamin B12 from the IOM are 2.4 μg/day, which is deemed appropriate for saturation divers. Circulating levels of folate are lowered after a saturation dive [9], and therefore it is suggested that folate intake corresponding to the RDA of 400 µg/day is appropriate [20] but higher intakes may be prudent. However, it is advised that intake does not exceed the recommended upper tolerable limit of 1000 μg/day [20]. Recommendations for each nutrient can be achieved through a varied diet; nevertheless, there may be some instances where supplementation may be required, in particular folate, if availability is insufficient from on-board catering provision.

#### Iron recommendations

Iron also has an important role in haematological processes. However, iron has been reported to accumulate within the body during saturation, which is likely to be due to the decline in red blood cell content [11]. Excessive iron can result in the formation of powerful ROS [60] and is associated with a greater risk of type 2 diabetes [61]. For this reason, we suggest dietary intake of iron should be monitored. Current dietary recommendations of iron are 18 mg/day, there is not sufficient evidence to lower this recommendation but saturation divers are not advised to increase intake beyond this or supplement with iron in the chamber.

### Hydration

Maintenance of fluid and electrolyte homeostasis is required to ensure usual physiological function, due to the wide range of functions which water fulfils within human metabolism [62]. Hypohydration occurs during the process of dehydration whereby fluid loss is greater than fluid intake, and is thought to cause a range of adverse health and performance-related implications [62, 63]. Many decrements are thought to commence at approximately 2 % body mass loss [63]. The hyperbaric hyperoxic environment of saturation diving induces a number of physiological changes, which may contribute to a state of hypohydration.

The primary factor that may challenge fluid homeostasis within the saturation chamber is hyperbaric diuresis. This refers to an excess production of urine, which has been reported to occur under conditions of hyperbaric hyperoxia [4–6]. Sagawa et al. [6] reported a significant twofold increase in daily urine production (1032 ± 140 to 2100 ± 105 ml, *p* < 0.05), a 54 % increase in sodium excretion (163 ± 24 to 251 ± 14 meq/24 h, *p* < 0.05) and a 32 % reduction in urine osmolality (799 ± 78 to 541 ± 29 mosmol, *p* < 0.05) during a 7-day simulated saturation dive. Furthermore, the increased sodium loss [6] may contribute to a state of hypovolemia (i.e. decreased plasma volume). This is supported through reported plasma volume reduction of 20 ± 9.3 and 30.1 ± 9.1 % at 450 and 230 m, respectively, found during a simulated hyperbaric dive [64].

In addition to the hydration challenges associated with the hyperbaric environment, underwater work periods may also compromise the body’s ability to maintain euhydration. Performing physically demanding work while immersed in water and wearing a hot water diving suit may create a state of thermal stress [65], which is likely to increase fluid loss due to increased sweat rate and production. A number of studies have reported that seawater immersion lasting between 4 and 6 h results in significant fluid loss [65–68] due to the hyperosmolarity of seawater, which exacerbates fluid loss. In a study evaluating body mass loss during lockout in 128 divers, Hope et al. [64] reported that subjects lost ≥2 % body mass during one-third of dives (corresponding to the threshold typically reported as resulting in performance decrement), with losses up to 6 % body mass loss reported, representing a state of severe dehydration [69].

#### Hydration recommendations

The ultimate goals of the hydration recommendations made are to encourage maintenance of in-chamber euhydration, and to ensure sufficient fluid replacement following lockout. Prescriptive recommendations related to hydration strategies are difficult due to the highly individual nature of the body’s response. In accordance with guidelines from the British Dietetics Association, a minimum of 2 L/day of water is recommended. This recommendation aligns with those provided for astronauts, a group who experience similar environmental challenges to saturation divers, including changes to atmospheric pressure, and confinement for prolonged periods of time.

Given the large fluid losses reported during lockout, along with the limited opportunities to drink, more extensive hydration strategies may be required in order to counteract these factors. The primary pre-lockout goal should be to ensure that divers enter lockout in a euhydrated state, with the consumption of food and fluid containing sodium to be used prior to lockout to facilitate fluid retention [70, 71]. Due to the iso-osmotic composition of fluid loss underwater [65], an isotonic electrolyte drink is recommended during a dive to replace electrolyte losses, thus reducing the likelihood of hyponatremia or large reductions in plasma volume. Post lockout, it may be advantageous to practice more extensive hydration strategies to facilitate rapid recovery in preparation for the next lockout period, typically within the next 12–24 h. Sodium intake in this instance is critical for the effective restoration of fluid balance within euhydration ranges [70, 71]. It is important to consider that these recommendations may inadvertently increase the risk of over-hydration and the development of hyponatremia, which are associated with adverse health [72]. As such, in the absence of direct measurements of fluid balance, divers are encouraged to dictate fluid intake with sensation of thirst when consuming fluids above the minimum 2 L recommended.

### Additional nutritional considerations

The aspects highlighted within these guidelines have primarily focused on the role of nutrition on the health and performance of saturation divers. There is, however, emerging evidence to suggest that nutritional strategies may also enhance safety of divers during decompression. Administration of exogenous nitric oxide (NO) reduces microbubble formation after decompression [73] in divers, whilst knockout of nitric oxide synthase (NOS) in mice increased microbubble formation [74]. The presence of gaseous microbubbles is positively correlated to the risk of decompression sickness [2], for that reason dietary strategies to increase nitric oxide availability for decompression may be beneficial. Manipulation of dietary intake can augment nitric oxide bioavailability through exogenous nitrate intake [75], which is predominantly obtained through vegetables such as spinach, beetroot and rhubarb. We recommend divers should be encouraged to consume a healthy diet rich in nitrates throughout a saturation dive. If practical barriers in catering provision prevent divers from obtaining dietary nitrates, supplementation with l-arginine can be used as an alternative to increase NO production [75]. Nevertheless, these recommendations are somewhat speculative given the limited scientific evidence in the area. Thus, further research is required to ascertain the clinical relevance of such recommendations. That said, dietary intake of nitrates is deemed a safe but potentially effective recommendation.

### Practical challenges and recommendations to nutritional intake

In addition to the physiological factors described above, there are also a number of practical challenges associated with saturation diving that may present barriers to achieving appropriate nutritional intake. Therefore, nutritional recommendations for this population should be cognisant of both physiological and practical considerations, thereby allowing greater opportunity to practice appropriate dietary strategies within this challenging environment. The greatest practical barriers to nutritional intake include shift patterns and food provision, lockout and food palatability. These areas will be discussed in more detail below, along with recommendations to facilitate nutritional intake to overcome practical barriers.

#### Shift patterns and food provision

Saturation divers are allocated 12 h shift patterns, during which they can be asked to enter lockout. This necessitates the adjustment of daily routines to the allocated time periods and may distort perception of normal night and day. It has been suggested that shift work (within the general population) can impact upon the ability to maintain usual eating patterns, particularly if food choices are limited [76]. At any given time of day, divers may have different dietary needs that will have implications for catering provision on board the vessel. As one diving team is returning from an intense underwater excursion, another team may be waking from sleep and desire breakfast food items. In this case, we suggest that catering providers work in conjunction with an appropriately qualified nutrition professional (e.g. dietitian or registered nutritionist) to develop menus that will enable saturation divers to meet their specific nutritional requirement regardless of shift allocation. On larger diving support vessels that may have eight diving teams on different shift patterns, this will require appropriate planning and communication between the catering providers, diving support team and nutritional professional.

#### Lockout

During lockout saturation, divers are isolated from the main vessel for up to 8 h, 6 of which may involve underwater immersion and the completion of physically demanding tasks whilst wearing a hot water diving suit. Previously, divers had limited opportunity to refuel or rehydrate during lockout, although recent legislative changes stipulate a mandatory break if they are to be immersed for 4 h or longer, which may have a positive influence on ability to maintain fluid and energy balance during lockout. Simple steps can be taken to encourage dietary intake in this situation, for example, provision of energy dense snacks in the bell may encourage intake. These snacks can be consumed while the divers are being transported to and from the working depth or during the mandatory 30-min break mid-lockout. The use of a carbohydrate-based sports drink may be most appropriate for this situation as it can provide the energy, fluid and electrolytes that are required when working. Sport nutrition research has shown that the minimum rate of carbohydrate ingestion to elicit physical performance benefits is 16 g/h [77], which is a suitable minimum recommendation for underwater excursions in order to maintain performance. Ingestion at higher levels, between 30 and 60 g/h, in line with current sports nutrition recommendations [78] should be based on the individual tolerance and the intensity of work undertaken. Previously, a water carriage system integrated within diving suits has been piloted with some success [79], providing a potentially suitable and effective method of energy intake and maintaining fluid balance while underwater; however, the use of such apparatus is thought to be limited amongst saturation divers due to debates over their practicality and safety underwater.

The timing of energy intake around lockout is also deemed pertinent. Increased energy availability has been suggested to have a considerable impact upon physical performance [80], more specifically, the consumption of carbohydrates and dietary fats 3–4 h prior to exercise is suggested to have an ergogenic impact on exercise performance [81]. Additionally, the restoration of muscle glycogen and intramuscular triglyceride stores following lockout may be beneficial, particularly if subsequent shifts are within the next 12–24 h. A meal rich in moderate to high glycaemic index carbohydrates, along with dietary fats, are recommended [82] post lockout to facilitate recovery. For this to be practiced, we recommend that the diving support and catering teams communicate appropriately, so food is available for each diver within a reasonable time of entering and leaving lockout. In addition, it is likely that the intensity and duration of an underwater excursion will be known prior to the event. Communication of the intended work schedule to the catering team will allow menus to be developed that provide the appropriate amount of energy for the proposed work. Where possible both teams should plan ahead, however this will require a certain degree of flexibility on behalf of the catering team as schedules are susceptible to last minute change. Effective implementation of this recommendation requires the use of a qualified nutrition professional to facilitate the communication, in order to ensure the energy expended during lockout is matched with the calories available in food provision, whether this is through energy dense meal options or varying portion sizes.

#### Food palatability and appetite

All food that enters the saturation chamber is compressed to the ambient pressure, which in certain instances appears to distort the perception of food palatability [18, 83]. Thorp and Doubt [83] recorded the perception of different foods at varying depths with results indicating that certain foods were distorted and not accepted for consumption by divers. Milk, for example, became warm and unpalatable, only fresh vegetables were found to maintain their taste, and meat had to be of good quality and cooked well to maintain palatability in the chamber. Little is known about the contributing factors to the taste and smell distortion, although it is likely a combination of factors originating from the increased pressure and the helium-rich environments. Further research to elucidate the contributing factors may be warranted given the sensory perception of food has a critical role in regulating appetite and dietary intake [84]. Along with taste and smell, sensory perception is influenced by the food texture and aesthetics, both aspects that can be controlled during food preparation. We recommend that the catering professionals on the vessel should be made aware of the significant role they have in promoting the health and performance of saturation divers and be encouraged to place emphasis on the texture and the visual presentation of the food.

Personal communication between the authors and saturation divers has also revealed that appetite may be suppressed after an intense shift underwater. This phenomenon warrants scientific investigation; however, the theory is plausible as participation in physically demanding activities has been reported to transiently reduce hunger sensations [85], representing an additional challenge to appropriate dietary intake. Due to this, we recommend that during incidences of suppressed appetite, the availability of a liquid based energy dense meal may be a suitable alternative.

## Summary and conclusions

This review aimed to provide nutritional recommendations for saturation divers based upon the physiological and practical challenges encountered by these individuals. The primary recommendations are summarised within Table 2 and these are based on an in-depth review of the available literature, along with consideration of the practical challenges associated with this occupation. It should be noted, however, that substantial gaps exist within the evidence base related to the physiological response of the body to prolonged confinement within a hyperbaric, hyperoxic environment, and much of the available literature is relatively dated and involved small sample sizes, limiting its generalisability to modern-day divers. In accordance with these limitations, the recommendations within this review are made with caution, and are within commonly accepted public health and athletic recommended ranges. This review provides practical, evidence-based guidance that may promote health, safety and performance within this challenging environment and occupation. Further research is required in order to better understand the physiological effect and its clinical significance to the long-term health and well-being of saturation divers, along with the potential of nutritional strategies to enhance health and performance within this group.

**Table 2:** Summary of nutritional recommendations

Energy requirements during a saturation dive are greater than the surface. We recommend saturation divers should consume between 44 – 53 kcal/Kg BM, dependent on the intensity and duration of subsea shift work. Energy dense foods and high-energy meal replacement supplements should be considered to meet the elevated energy expenditure, particularly if periods of suppressed appetite are experienced post lockout
Protein intakes of at least 1.3 g/kg BM are recommended to support diminished WBPS and reduce periods of catabolism
Fat and carbohydrate intake are essential for achieving energy balance. We recommend the percentage of total calorie intake from dietary fat should be at the top of end of the current IOM guidelines (20-35 %), whilst carbohydrate intake should be at the lower end of IOM recommendations (45-65 %). Despite this we suggest that fat and carbohydrate intake should ultimately be dictated by personal preference to attain energy balance
A varied diet high in fruit and vegetables are recommended for achieving micronutrient targets. More specifically, antioxidants, vitamin B12 and folate are of greater interest due to their respective involvement in redox physiology and red blood cell production
Supplementation with Vitamin D is recommended due to the absence of UVB ray exposure
A diet rich in dietary nitrates may enhance decompression safety through attenuating gaseous microbubble formation
Prescriptive recommendations for hydration are difficult due to large inter-individual differences in fluid and electrolyte homeostasis. The challenge for saturation divers is twofold; that of hyperbaric diuresis and prolonged underwater immersion. The addition of sodium within food and beverages may facilitate fluid and electrolyte restoration and maintenance of homeostasis
Meals rich in carbohydrates and fats are recommended prior to and proceeding lockout to increase energy availability for underwater work and enhance recovery post lockout. Consumption of a carbohydrate-based beverage, at a minimum of 16 g/hr, during lockout may also support physical performance underwater
Catering providers have a pivotal role in facilitating appropriate nutritional intake. Catering professionals should be encouraged to place emphasis on the food texture and presentation to increase the sensory appeal of foods in the chamber, thereby supporting increased energy intake

## Abbreviations

BM: Body mass
DCS: Decompression sickness
EPO: Erythropoietin
GPx: Glutathione peroxidise
IOM: Institute of Medicine
Kcal: Kilocalorie
NO: Nitric oxide
NOS: Nitric oxide synthase
PPO_2_: Partial pressure of oxygen
RER: Respiratory exchange ratio
RDA: Recommended daily allowance
ROS: Reactive oxygen species
SOD: Superoxide dismutase
UVB: Ultraviolet B
WBPS: Whole body protein synthesis
